# Enhanced Heat-Electric Conversion via Photonic-Assisted Radiative Cooling

**DOI:** 10.3390/nano11040983

**Published:** 2021-04-11

**Authors:** Jeng-Yi Lee, Chih-Ming Wang, Chieh-Lun Chi, Sheng-Rui Wu, Ya-Xun Lin, Mao-Kuo Wei, Chu-Hsuan Lin

**Affiliations:** 1 Department of Opto-Electronic Engineering, National Dong Hwa University, Hualien 97401, Taiwan; jengyilee@gms.ndhu.edu.tw (J.-Y.L.); 610725011@gms.ndhu.edu.tw (C.-L.C.); 2 Department of Optics and Photonics, National Central University, Taoyuan 32001, Taiwan; 410325015@gms.ndhu.edu.tw; 3 Department of Materials Science and Engineering, National Dong Hwa University, Hualien 97401, Taiwan; yaxunlin@gapp.nthu.edu.tw (Y.-X.L.); mkwei@gms.ndhu.edu.tw (M.-K.W.)

**Keywords:** radiative cooling, Seebeck effect, thermoelectric generator (TEG)

## Abstract

In this paper, an inorganic polymer composite film is proposed as an effective radiative cooling device. The inherent absorption is enhanced by choosing an appropriately sized SiO_2_ microsphere with a diameter of 6 μm. The overall absorption at the transparent window of the atmosphere is higher than 90%, as the concentration of SiO_2_–PMMA composite is 35 wt%. As a result, an effective radiative device is made by a spin coating process. Moreover, the device is stacked on the cold side of a thermoelectric generator chip. It is found that the temperature gradient can be increased via the effective radiative cooling process. An enhanced Seebeck effect is observed, and the corresponding output current can be enhanced 1.67-fold via the photonic-assisted radiative cooling.

## 1. Introduction

At present, most of the main energy sources convert petrochemical or nuclear energy into kinetic energy, which is then converted to electric power by a bulky mechanical electric generator. Photovoltaic (PV) energy harvesting technology directly converts solar power to electricity without an electric generator. Moreover, owing to the compact size of the PV panel compared to the bulky mechanical electric generator, versatile applications that traditional electricity systems cannot offer emerge. Consequently, converting ambient energy, for example, solar power and a heat source, to electric power to operate low-power electronic devices or replace small batteries is an alternative potential application of green energy technology via a small power generation device. Nevertheless, the theoretical efficiency of a PV cell is limited by the matching spectrum between the solar and solar cell. Consequently, some energy up-conversion concepts, such as thermophotovoltaic [1] and multiphoton absorption [2], have been proposed to convert the long-wavelength energy to short-wavelength energy in order to improve the matching spectrum for a higher PV conversion efficiency. Thermal-to-electricity conversion technology harvests heat via a thermoelectric generator (TEG) chip with a size similar to a PV cell. Compared to commonly used PV energy harvesting technology, thermal-to-electricity conversion is not limited by the weather. Therefore, a TEG is more suitable and stable than a PV cell to harvest ambient energy. 

In 1977, Bartoli et al. first demonstrated cooling technology based on thermal radiation [3]. Radiative cooling is the natural heat transportation process through which objects shed heat in the form of radiation. All objects at room temperature emit heat via radiation. As the emission wavelength of an object is tuned to be at the transparency window (8 μm to 14 μm) of the atmosphere, the heat can radiate to the cold sink of outer space via the transparency window of the atmosphere. This technology is called radiative cooling. A number of works aimed at designing various photonic and plasmonic structures for radiative cooling applications have also been proposed [4,5,6,7,8]. For example, based on the metal–dielectric photonic structure, E. Rephaeli et al. first demonstrated a net cooling power in excess of 100 W/m^2^ at ambient temperature [4]. A.P. Raman et al. experimentally demonstrated that the HfO_2_/SiO_2_ multilayer photonic radiative cooler cools to 4.9 °C below the ambient temperature under direct sunlight illumination and 19.5 °C lower than the ambient temperature at night [9]. By using a visibly transparent silica photonic crystal, L. Zhu et al. experimentally demonstrated that the temperature of a silicon absorber under sunlight can be reduced by 13 °C due to radiative cooling [10].

For practical applications, in particular, low-cost passive radiative cooling with a large device area is of much interest. Therefore, owing to the relatively simple and low-cost fabrication process, radiative cooling based on micro-/nanoparticles attracts a great deal of research interest. For example, radiative coolers based on SiC/SiO_2_ nanoparticle composites [5], SiO_2_ particle embedded poly(4-methyl-1-pentyne) (TPX) film [7], and SiO_2_ microsphere white paint [11] are all easy to produce at a low cost and present the capability to exceed 10 °C below ambient temperature.

## 2. Materials and Methods

We know that the sun is the largest provider of green energy. When we harvest solar energy, we need a device to absorb solar light and then convert the solar energy to different forms of energy—for example, converting solar energy to electric potential such as solar cells, converting solar energy to chemical potential, and converting solar to thermal energy. Regardless, as we harvest solar energy, conversion loss is unavoidable, and heat is eventually generated, which means that the Earth will be heated. Consequently, the surfaces of current high-efficiency solar panels are black to absorb a wide spectral range of sunlight. However, only a fraction (usually below 20% for commercially available solar panels) of this incoming energy is converted to electric power. The rest is returned to the environment as heat. The solar panels are usually much darker than the ground. As a result, the solar panels absorb a great deal of additional solar energy, and the Earth is thus heated.

**Figure 1:**
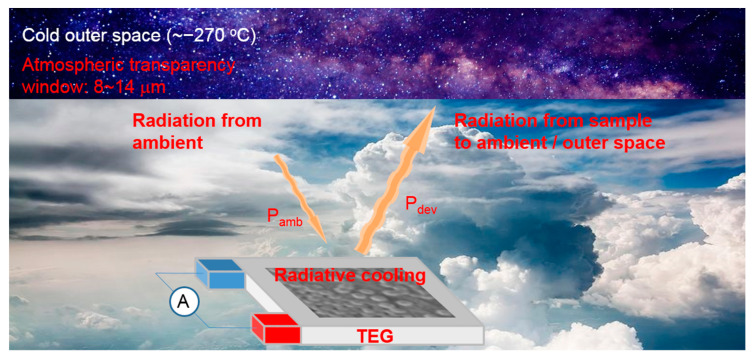
Conceptual schematic of the proposed device for harvesting ambient energy via passive radiative cooling device.

In this paper, we would like to demonstrate a method to harvest the ambient energy by returning the heat to outer and generating electric energy. The concept of the proposed device is shown in Figure 1. Radiative cooling technology is utilized to create a temperature gradient on a TEG chip by radiating the ambient heat to outer space. Via the photonic-assisted radiative cooling, the enhancement of the Seebeck effect of the TEG chip is observed. The heat will be sent to outer space but will not be absorbed. Thus, we are able to harvest an inexhaustible supply of ambient heat energy. Therefore, the Earth will not be heated. We believe that our proposed method is greener than the current green energy.

The device consists of a TEG chip and a passive radiative cooling device. The TEG chip with a device area of 15 mm × 15 mm is composed of two dissimilar thermoelectric materials, a *p*-type and an *n*-type semiconductor, connected at their ends. The temperature gradient in the thermoelectric material leads to free carrier diffusion, so that a voltage difference between the hot and cold sides of the TEG can be created. The corresponding power, *P_TEG_*, delivered to an external load is [12]:

(1) $$P_{TEG}=\frac{S^{2}\Delta T^{2}R_{e}}{{\left(R_{TE}+R_{e}\right)}^{2}}$$

where *R_TE_* and *R_e_* are the electric resistance of the TEG and the external load resistance, respectively. *S* is the Seebeck coefficient, and Δ*T* is the temperature difference across the TEG. It can be seen that the output power is proportional to Δ*T*^2^. Therefore, we can increase the power that is driven from the TEG by maximizing the temperature gradient. Here, we utilize a passive radiative cooling device to direct the environmental heat toward outer space so that the temperature gradient between the hot and cold sides of the TEG can be increased. Consequently, we are able to enhance the output power of the TEG.

**Figure 2:**
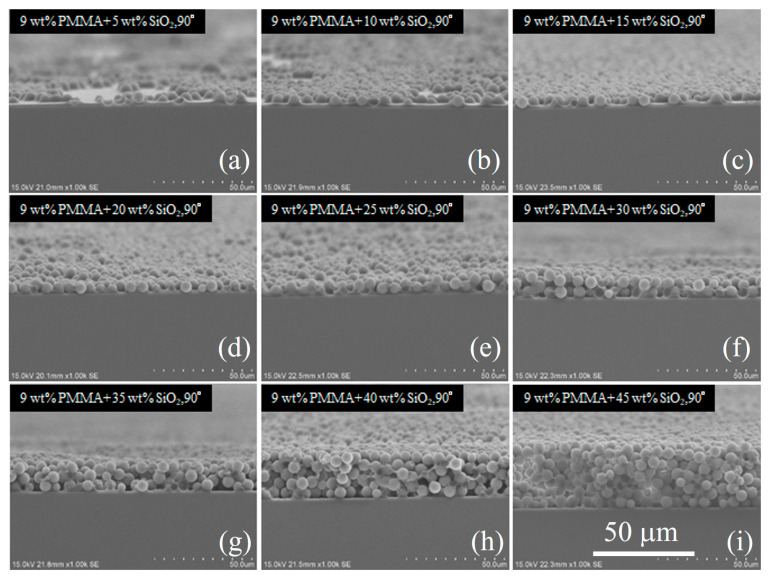
Side-view of the SEM photograph of the fabricated SiO_2_–PMMA composite film with a total area of 15 mm × 15 mm. (**a**–**i**) indicate the weight percent concentration of SiO_2_ particles gradually increased from 5 wt% to 45 wt%.

Recently, inorganic polymer nanocomposites have attracted significant interest as emerging materials due to their unique combination of properties as compared to pure polymers [13,14]. The physical properties of the composites can be easily modified via changing the compositions and concentrations. Here, we selected SiO_2_–PMMA composites to fabricate a radiative cooling device. The absorption of the nanocomposite film can be tuned by simply modifying the concentration of SiO_2_. SiO_2_ nanoparticles were dispersed and mixed in PMMA for nine different concentrations (5, 10, 15, 20, 25, 30, 35, 40, and 45 wt%). The SiO_2_ microparticles are commercially available. They were purchased from Promagic Technology Co., Ltd. (Taoyuan, Taiwan). The product name was PMG-5506. The average diameter of the SiO_2_ particles was 6 μm. The SiO_2_–PMMA composite was then spin-coated onto a Cu substrate. The reason that we chose Cu was the possibility of achieving heat conduction between the radiative cooling film and the cold side of the TEG chip. A side view of the scanning electron microscope (SEM) photograph of the spin-coated SiO_2_–PMMA composite film is provided in Figure 2. The PMMA weight percent was 9 wt%. Under this concentration, the SiO_2_ particles can be successfully adhered. The optical properties mainly depend on the SiO_2_ microsphere. Figure 2a–i indicate the weight percent concentration of the SiO_2_ particle, which gradually increased from 5 wt% to 45 wt%. It can be seen that the SiO_2_ dispersed uniformly, and most of the area was covered by a single layer of SiO_2_ particles as the SiO_2_ concentration ranged from 15 wt% to 25 wt%. A higher weight percentage of SiO_2_ leads to a higher filling ratio. Consequently, a higher filling ratio of SiO_2_ spheres leads to higher absorption, which will be discussed in the following modeling section. When the SiO_2_ concentration is lower than 10 wt%, the substrate is not fully covered by SiO_2_ particles. When the SiO_2_ concentration is 30 wt% and 35 wt%, it presents two layers of closely packed SiO_2_ particles. When the SiO_2_ concentration is higher than 40 wt%, it presents multiple layers of closely packed SiO_2_ particles.

For an effective radiative cooling device, it is necessary to enhance the emissivity at the transparent window of the atmosphere. The heat at this spectral range can effectively radiate to the cold sink of outer space so that the heat can be directed to outer space. Additionally, it is necessary to suppress the absorption of the sideband, specifically the solar spectral range. The sideband refers to the spectral range that is outside of the transparent window of the atmosphere. By doing this, the device will emit the heat into the environment without absorbing it back again. The absorption spectrum of an ideal radiative cooling device is 100% at the transparent window of the atmosphere and is 0% at all others [15]. Both the PMMA and SiO_2_ are transparent, with a broad bandwidth, as is the absorption at the transparent window of the atmosphere. Additionally, the SiO_2_ supports both dipole-allowed transverse optical (TO) vibration and longitudinal optical (LO) bond-bending vibration modes at 9 μm and 12 μm, respectively. The phonon absorption bandwidth of SiO_2_ is fully covered by the spectral range of the transparent window. Therefore, SiO_2_–PMMA inorganic polymer nanocomposites are a potential candidate for radiative cooling applications because of the transparency at the visible range and the high absorptivity at the transparent window. For lower absorption in the visible spectral range, an Al substrate might be a better choice than Cu. However, the heat conduction of Cu is much better than that of Al. Therefore, a trade-off exists between the reflectivity and conductivity of a metal substrate.

As mentioned, the diameter of the utilized SiO_2_ particles is 6 μm. As the spin-coated SiO_2_ particles are closely packed, the periodicity of the SiO_2_ particle array is identical to its diameter. At this time, according to classic diffraction theory, the diffraction angle is close to 90° for normally incident light with the wavelength of the transparent window of the atmosphere. Additionally, the Fourier transformation of the SiO_2_ sphere is a ripple-like function consisting of spherical Hankel functions [16]. As spherical particles are closely packed, the lattice is hexagonal. As we consider the Γ point of the reciprocal space, the period (Λ) is the diameter of the SiO_2_ microparticles. The period, 6 μm, is approximately half of the transparent window’s wavelength (8–14 μm). Under this condition, all the diffraction orders in free space are evanescent. However, there is still a possibility that the light can be coupled within the closely packed SiO_2_ microparticles. At this time, the absorption path of the coupled light can be dramatically prolonged, and the emissivity can be thus increased. This phenomenon is called the guided-mode resonance effect [17].

**Figure 3:**
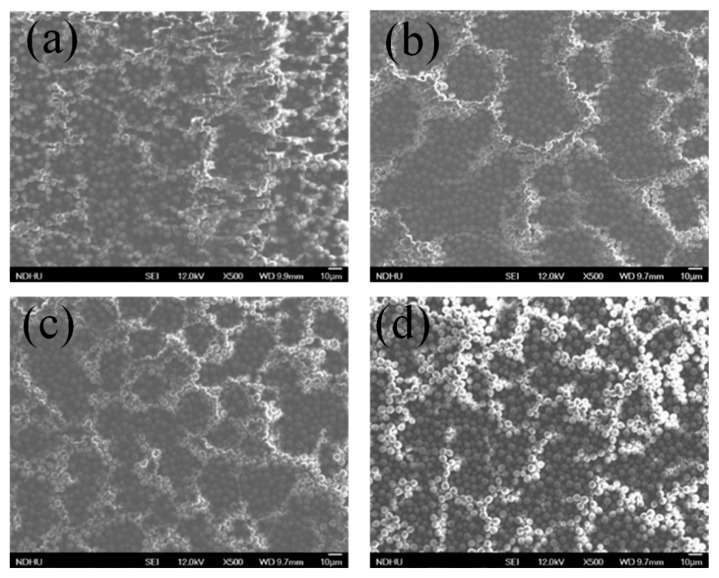
Top-view SEM photograph of the fabricated SiO_2_–PMMA composite film for the SiO_2_ particle weight percent concentrations of (**a**) 15 wt%, (**b**) 20 wt%, (**c**) 25 wt% and (**d**) 35 wt%.

The top-view of the SEM photograph of the fabricated SiO_2_–PMMA composite film for the SiO_2_ particle with different weight percent concentrations is provided in Figure 3. As shown in Figure 3a, for 15 wt%, some areas of the spin-coated SiO_2_–PMMA composite are not closely packed. As the weight percent concentration of the SiO_2_ particle is 20 wt% (Figure 3b), the SEM photograph reveals that the SiO_2_ microparticles are closely packed, which is consistent with the side-view SEM. For 25 wt% (Figure 3c), it can be seen that the SiO_2_ stacks as a multilayer structure.

Measurement of the absorption was performed with a micro-Fourier transform infrared spectroscopy with a reflective-type microscope objective with a magnification of 36×. A liquid-N_2_-cooled HgCdTe infrared detector was used for the detection of radiation.

## 3. Results and Discussion

**Figure 4:**
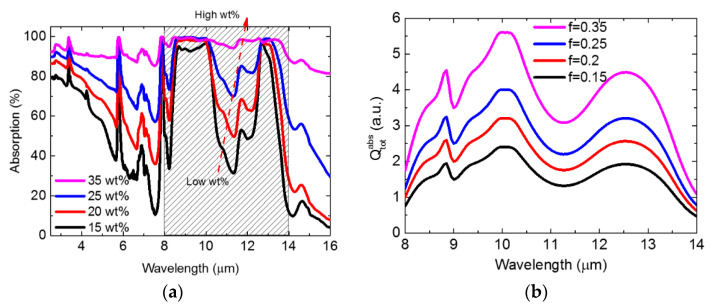
(**a**) Absorption spectra of the SiO_2_–PMMA nanocomposite film. Black, red, blue, and pink lines represent the absorption spectrum of 15 wt%, 20 wt%, 25 wt%, and 35 wt% SiO_2_–PMMA composite films, respectively. (**b**) Resultant powers of collective incoherent SiO_2_ particles calculated by Equation (2). Here, the spectrum that we show is consistent with the atmospheric transparency window, and the material dispersion of SiO_2_ is based on experimental data. The total powers for 15 wt%, 20 wt%, 25 wt%, and 35 wt% SiO_2_–PMMA composite films are depicted in black, red, blue, and pink lines, respectively. The shadow in Figure 4a indicates the region of transparency window.

The absorption spectrum of the SiO_2_–PMMA composite film is provided in Figure 4a. Black, red, blue, and pink lines represent the absorption spectra of the 15 wt%, 20 wt%, 25 wt%, and 35 wt% SiO_2_–PMMA composite films, respectively. For the radiative cooling application, the spectral range from 8 μm to 14 μm is of particular concern. In this range, for the 15 wt% sample, it can be seen that absorption dips at 11.5 μm, which corresponds to the phonon absorption of SiO_2_. As the concentration increases, the absorption gradually increases. As shown in Figure 4a, for the 35 wt% sample, the overall absorption at the transparent window is higher than 90%. For the 40 wt% and the 45 wt% samples, the absorption spectrum is very similar to the results of the 30 wt% sample. This is because the SiO_2_ particles stack as a multilayer structure as the concentration is higher than 35%. Additional layers of SiO_2_ particles do not enhance the absorption significantly. Therefore, we did not show the spectrum for the 40 wt% and the 45 wt% samples. The spectrum revealed high absorption from 8 μm to 14 μm. According to Kirchhoff’s law of thermal radiation, the emissivity at this range is high and is beneficial for radiative cooling. Therefore, the 35 wt% sample is more suitable as a radiative cooling device as compared to the others. 

Next, to understand the underlying mechanism behind the measured results given in Figure 4a, we calculated the total absorption of SiO_2_ while taking the total number effect into account. In [18,19], the authors theoretically proved that for a finite-sized object, its thermal emission is proportional to the absorption cross-section. Here, we model SiO_2_ as a spherical scatterer and investigate its resultant absorption within the atmospheric transparency window. Furthermore, based on exact Mie’s scattering theory [20], as well as considering the total number effect, the total absorption cross-section $Q_{tot}^{abs}$ can be estimated as follows (see [21]).

(2) $$Q_{tot}^{abs}=N\times Q_{abs}=\frac{M_{SiO_{2}}}{\frac{4\pi }{3}a^{3}\rho }Q_{abs}\propto fQ_{abs}$$

Here, *N* is the total number of SiO_2_ particles, and *Q_abs_* is the absorption cross-section by a single SiO_2_ particle, $M_{SiO_{2}}$ is mass, *a* is size, *ρ* is the mass density of SiO_2_, and *f* is weight ratio. This simple expression reveals that increasing the weight ratio of SiO_2_ could directly enhance the resultant absorption. Figure 4b demonstrates that the system with a concentration 35 wt% can harvest more absorption power in this transparency window, implying the higher emission compared to other schemes. Notably, in the range of 11 μm, the degradation of absorption stemming from the phonon resonant effect can be also observed in Figure 4b. In addition, another dip signature of absorption at 9 μm, shown in Figure 4a, is also evidenced by our calculation results. Our numerical calculation demonstrates good agreement with the experiments shown in Figure 4a.

The directional spectral absorptivity, *α_dev_*(*T_dev_*, *λ*, *θ*), was measured using FTIR. As radiative cooling samples consisting of materials satisfy Lorentz reciprocity [22], the general form of Kirchhoff’s law states that the directional spectral emissivity, *ε_dev_*(*T_dev_*, *λ*, *θ*), is equal to the directional spectral absorptivity. Here, we simply assume that the absorption in the dependence of observing angles is averaged in angles owing to the nature of the microscope measurement setup. Therefore, the emissivity of the SiO_2_–PMMA nanocomposite film as a function of wavelength and observation angle can be obtained based on the measured absorption spectrum shown in Figure 4. Moreover, the change in the emissivity can be ignored within the considered temperature range from 20 °C to 60 °C. Therefore, in practice, only *ε_dev_*(*T_dev_*, *λ*, *θ*) at room temperature is measured. Integrating the product of the directional spectral emissivity and temperature-dependent spectral radiance of a blackbody, *U_B_*, which can be calculated from Planck’s law, one can obtain the theoretical radiation power density (*P_dev_*) of the device:

(3) $$P_{dev}=\int_{0}^{\pi /2}\pi \sin2\theta \;d\theta \;\int_{0}^{\infty }U_{B}\left(T_{dev},\;\lambda \right)\;\varepsilon_{dev}\left(T_{dev},\;\lambda ,\;\theta \right)d\lambda \;$$

Here, we assume that the heat transfer is based on thermal radiation only. The heat transfer via convection and conduction is ignored. The heat radiates outside the atmospheric transparency window and parasitically absorbs heat radiation from the atmosphere. Under this assumption, the net cooling power density, *P_net_*, is given by:

(4) $$P_{net}=P_{dev}-P_{amb}$$

where *P_amb_* denotes the radiation power density of the ambient atmosphere, respectively. Similar to the *P_dev_*, the *P_amb_* is also a function of the temperature and emissivity of the atmosphere. The emissivity of the atmosphere is taken from reference [23]. The atmospheric transmittance is nearly constant across all angles other than near the horizon [24]. Therefore, we simply assume that the emissivity of the atmosphere is independent of the angles.

**Figure 5:**
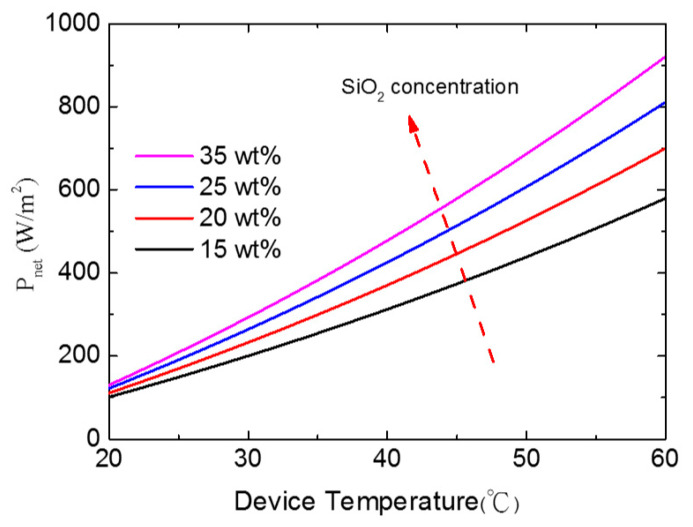
Theoretical net cooling power of the SiO_2_–PMMA nanocomposite film as a function of the radiative cooling device temperature. The ambient temperature is assumed to be 27 °C.

The *P_net_* of the SiO_2_–PMMA nanocomposite film as a function of the device temperature (*T_dev_*) is shown in Figure 5. The ambient temperature is assumed to be 27 °C. Black, red, blue, and pink lines represent the *P_net_* of 15 wt%, 20 wt%, 25 wt%, and 35 wt% SiO_2_–PMMA composite films, respectively. As the device temperature increases, *P_net_* almost linearly increases. A higher concentration of SiO_2_ leads to a higher *P_net_*. For *T_dev_* = 60 °C, *P_net_* of the 35 wt% sample (920 W/m^2^) is 1.67-fold higher than that of the 15 wt% sample (580 W/m^2^) owing to the high absorption of the high SiO_2_ concentration.

**Figure 6:**
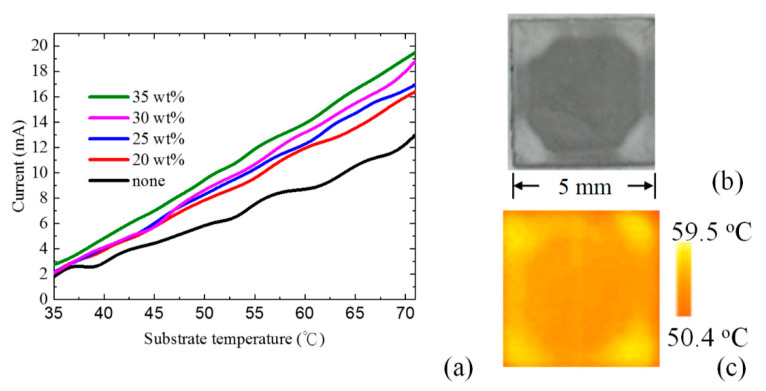
(**a**) Enhanced output current of thermoelectric generator (TEG) chip as a function of substrate temperature. Black line is the output current of the bare TEG chip. Red, blue, pink, and green lines represent the TEG stacked with the 15 wt%, 20 wt%, 25 wt%, and 35 wt% SiO_2_–PMMA composite films, respectively; (**b**) Photograph of the SiO_2_–PMMA composite film; (**c**) Temperature distribution of SiO_2_–PMMA composite film on a heated substrate.

Here, we utilize a hot plate as an ambient heat source. Figure 6a shows the output current of a TEG chip as a function of hot plate temperature. The hot side of the TEG chip is stacked on a hot plate with a temperature higher than the ambient temperature (27 °C, which is a common and recommended indoor temperature). The output current is the short circuit current measured after the chip is placed under thermally equivalent conditions. The black line indicates the output current of a bare TEG chip for reference. The red, blue, pink, and green lines indicate the output current of the TEG stacked with 20 wt%, 25 wt%, 30 wt%, and 35 wt% SiO_2_–PMMA sample, respectively, on the cold side. It is demonstrated that the output current can be slightly enhanced from 6 mA to 10 mA when the hot plate temperature is 50 °C. As mentioned, the phonon absorption of SiO_2_ is well-matched with the transparent window. According to Wien’s displacement law, the thermal emission peak of a blackbody is 8.97 μm at 50 °C. This thermal emission peak is also well-matched with the TO phonon absorption of SiO_2_. Therefore, the proposed cooling device is efficient at this temperature range—for example, a cup of hot coffee and an operating electronic device.

According to the Stefan–Boltzmann Law, the radiation flux increases with increasing substrate temperature. Consequently, *P_dev_* increases with the same trend. As shown in Figure 5, a higher SiO_2_–PMMA concentration leads to a higher emissivity at the window spectral range. Therefore, for a higher substrate temperature, the TEG holds a higher output current. Additionally, a higher-concentration sample holds a higher slope (output current to substrate temperature) owing to the corresponding higher emissivity. Nevertheless, here, we discuss the case in which heat transportation is radiation-dominated. Figure 6b,c present a photograph and the temperature distribution image of the SiO_2_–PMMA composite film on a heated substrate. The temperature distribution image was taken by using an FLIR thermal image camera. In the photograph in Figure 6b, different colors at the edge and at the center of the cooling chip can be observed. The SEM picture reveals that this is because of the stacking layers of the SiO_2_ microsphere. It stacks more layers of SiO_2_ at the edge than that at the center. This is a very common condition in spin coating. The temperature distribution image shown in Figure 6b reveals that the temperature at the edge is higher than that at the center. In 2018, T. Suichi et al. already proposed a radiative cooler based on SiO_2_–PMMA composites. However, they used three-layer SiO_2_-PMMA with an Al substrate [25]. Our experimental results reveal that the multilayer SiO_2_ blocks the heat conduction and heat convection. Therefore, the multilayer SiO_2_ structure suffers from high thermal conduction resistance. At this time, heat transportation is limited by the heat conduction and convection. Therefore, the temperature at the edge of the chip is higher than that at the center.

## 4. Conclusions

In summary, a cost-effective inorganic-polymer nanocomposite, SiO_2_–PMMA, is proposed as an effective passive radiative cooling device. The SiO_2_–PMMA film holds a broad transparent spectral range and high emissivity at the transparent window of the atmosphere. Additionally, the scattering field of the SiO_2_ microsphere indicates that the absorption path can be prolonged. The emissivity of the SiO_2_–PMMA can be modified via modifying the concentration of the SiO_2_ microsphere. Our total absorption calculations of SiO_2_ based on exact Mie theory offer a good explanation to accompany the experimental results. Although, in our study, the size of the SiO_2_ scatterer is fixed, we should note that by ensuring a proper system design by tuning the geometry, one can excite the absorption cross-section resonances, further leading to high net cooling power density. Using this photonic-assisted radiative cooling process, the cooling device passively provides a higher temperature gradient. By stacking the cooling device on the cold side of a TEG device, it is demonstrated that the output current of the TEG can be enhanced from 6 mA to 10 mA when the hot plate temperature is 50 °C. We demonstrate the harvesting of energy by returning the heat to outer space and generating electric energy, which is a cost-effective and new green technology.

## References

[1] Bauer T. (2011). Thermophotovoltaics: Basic Principles and Critical Aspects of System Design.

[2] Asahi S., Teranishi H., Kusaki K., Kaizu T., Kita T. (2017). Two-step photon up-conversion solar cells. Nat. Commun..

[3] Bartoli B., Catalanotti S., Coluzzi B., Cuomo V., Silvestrini V., Troise G. (1977). Nocturnal and diurnal performances of selective radiators. Appl. Energy.

[4] Rephaeli E., Raman A., Fan S. (2013). Ultrabroadband Photonic Structures to Achieve High-Performance Daytime Radiative Cooling. Nano Lett..

[5] Gentle A.R., Smith G.B. (2010). Radiative Heat Pumping from the Earth using Surface Phonon Resonant Nanoparticles. Nano Lett..

[6] Hossain M., Jia B., Gu M. (2015). A Metamaterial Emitter for Highly Efficient Radiative Cooling. Adv. Opt. Mater..

[7] Zhai Y., Ma Y., David S.N., Zhao D., Lou R., Tan G., Yang R., Yin X. (2017). Scalable-manufactured randomized glass-polymer hybrid metamaterial for daytime radiative cooling. Science.

[8] Wu S.-R., Lai K.-L., Wang C.-M. (2018). Passive temperature control based on a phase change metasurface. Sci. Rep..

[9] Raman A.P., Anoma M.A., Zhu L., Rephaeli E., Fan S. (2014). Passive radiative cooling below ambient air temperature under direct sunlight. Nature.

[10] Zhu L., Raman A.P., Fan S. (2015). Radiative cooling of solar absorbers using a visibly transparent photonic crystal thermal blackbody. Proc. Natl. Acad. Sci. USA.

[11] Atiganyanun S., Plumley J.B., Han S.J., Hsu K., Cytrynbaum J., Peng T.L., Han S.M. (2018). Effective Radiative Cooling by Paint-Format Microsphere-Based Photonic Random Media. ACS Photonics.

[12] Chen W.-H., Wu P.-H., Wang X.-D., Lin Y.-L. (2016). Power output and efficiency of a thermoelectric generator under temperature control. Energy Convers. Manag..

[13] Elim H.I., Cai B., Kurata Y., Sugihara O., Kaino T., Adschiri T., Chu A.-L., Kambe N. (2009). Refractive Index Control and Rayleigh Scattering Properties of Transparent TiO2Nanohybrid Polymer. J. Phys. Chem. B.

[14] Lü C., Guan C., Liu Y., Cheng A.Y., Yang B. (2005). PbS/Polymer Nanocomposite Optical Materials with High Refractive Index. Chem. Mater..

[15] Zhao B., Hu M., Ao X., Chen N., Pei G. (2019). Radiative cooling: A review of fundamentals, materials, applications and prospects. Appl. Energy.

[16] Ganic D., Gan X., Gu M. (2002). Three-dimensional evanescent wave scattering by dielectric particles. Optik.

[17] Lin S.F., Wang C.M., Tsai Y.L., Ding T.J., Yang T.H., Chen W.Y., Yeh S.F., Chang J.Y. (2013). A model for fast predicting and optimizing the sensitivity of surface-relief guided mode resonance sensors. Sens. Actuators B Chem..

[18] Greffet J.-J., Bouchon P., Brucoli G., Sakat E., Marquier F. (2016). Generalized Kirchhoff law. arXiv.

[19] Kattawar G.W., Eisner M. (1970). Radiation from a Homogeneous Isothermal Sphere. Appl. Opt..

[20] Bohren C.F., Huffman D.R. (1998). Absorption and Scattering of Light by Small Particles.

[21] Lee J.-Y., Tsai M.-C., Chen P.-C., Chen T.-T., Chan K.-L., Lee C.-Y., Lee R.-K. (2015). Thickness Effects on Light Absorption and Scattering for Nanoparticles in the Shape of Hollow Spheres. J. Phys. Chem. C.

[22] Kruger M., Bimonte G., Emig T., Kardar M. (2012). Trace formulae for non-equilibrium Casimir interactions, heat radiation and heat transfer for arbitrary objects. Phys. Rev. B.

[23] IR Transmission Spectra, Gemini Observatory Kernel Description. http://www.gemini.edu/?q/node/10789.

[24] Bhatia B., Leroy A., Shen Y., Zhao L., Gianello M., Li D., Gu T., Hu J., Soljačić M., Wang E.N. (2018). Passive directional sub-ambient daytime radiative cooling. Nat. Commun..

[25] Suichi T., Ishikawa A., Hayashi Y., Tsuruta K. (2018). Performance limit of daytime radiative cooling in warm humid environment. AIP Adv..

